# A Tendon Cell Specific RNAi Screen Reveals Novel Candidates Essential for Muscle Tendon Interaction

**DOI:** 10.1371/journal.pone.0140976

**Published:** 2015-10-21

**Authors:** Prabhat Tiwari, Arun Kumar, Rudra Nayan Das, Vivek Malhotra, K. VijayRaghavan

**Affiliations:** 1 National Centre for Biological Sciences-Tata Institute of Fundamental Research, Bangalore, India; 2 Centre for Genomic Regulation, Barcelona, Spain; UMR CNRS 5242 - ENS de Lyon- Université Lyon 1, FRANCE

## Abstract

Tendons are fibrous connective tissue which connect muscles to the skeletal elements thus acting as passive transmitters of force during locomotion and provide appropriate body posture. Tendon-derived cues, albeit poorly understood, are necessary for proper muscle guidance and attachment during development. In the present study, we used dorsal longitudinal muscles of *Drosophila* and their tendon attachment sites to unravel the molecular nature of interactions between muscles and tendons. We performed a genetic screen using RNAi-mediated knockdown in tendon cells to find out molecular players involved in the formation and maintenance of myotendinous junction and found 21 candidates out of 2507 RNAi lines screened. Of these, 19 were novel molecules in context of myotendinous system. Integrin-βPS and Talin, picked as candidates in this screen, are known to play important role in the cell-cell interaction and myotendinous junction formation validating our screen. We have found candidates with enzymatic function, transcription activity, cell adhesion, protein folding and intracellular transport function. Tango1, an ER exit protein involved in collagen secretion was identified as a candidate molecule involved in the formation of myotendinous junction. Tango1 knockdown was found to affect development of muscle attachment sites and formation of myotendinous junction. Tango1 was also found to be involved in secretion of Viking (Collagen type IV) and BM-40 from hemocytes and fat cells.

## Introduction

Mechanisms underlying the development of the myotendinous system in *Drosophila* and vertebrates are conserved at the cellular and molecular level. Interactions between muscles and tendons are necessary for their development and patterning [1,2]. In the *Drosophila* embryo, tendon cell precursors that do not interact with myofibres lose their identity and in adult fly, muscles degenerate after detachment from the tendon cells [3,4]. Similarly, studies in avian limb have shown that in absence of myotendinous interactions, muscles and tendon cells degenerate [5,6]. Given these similarities, *Drosophila* muscle-tendon cell junction can be used as a model for the study of the myotendinous development and maintenance. Moreover, *Drosophila* indirect flight muscles (IFMs) show structural similarity to vertebrate muscles, where many myofibres are present in one muscle bundle [7]. Thus studying the adult *Drosophila* myotendinous system is of particular interest as it provides insight into the understanding of several myopathies and tendinopathies; beside these aspects, it also provides insight into cell-cell interactions which are of general interest to cell biology.

*Drosophila* adult muscle precursors are specified in the embryo in response to Notch signaling and later proliferate under regulation of Wingless signaling on wing disc notum [8,9]. Adult myogenesis is initiated at the onset of pupation when larval muscles histolyze and adult muscles start to develop. In *Drosophila* larvae three oblique muscles in the second thoracic segment called dorsal oblique muscles do not undergo histolysis during pupation; instead these templates grow by fusion of adult-specific myoblasts and form dorsal longitudinal muscles (DLMs) [10,11,12]. The developing DLMs provide a useful model to identify genes required for myotendinous junction (MTJ) formation because the larval templates as well as the developing DLMs can be visualized by a GFP fusion protein with myosin heavy chain (MHC-tau-GFP) [13]. Development of DLMs is well characterized and easily manipulated by genetic perturbations which, depending upon the severity, can lead to pupal lethality, flight defects or visible defects in formation of MTJs.

**Fig 1:**
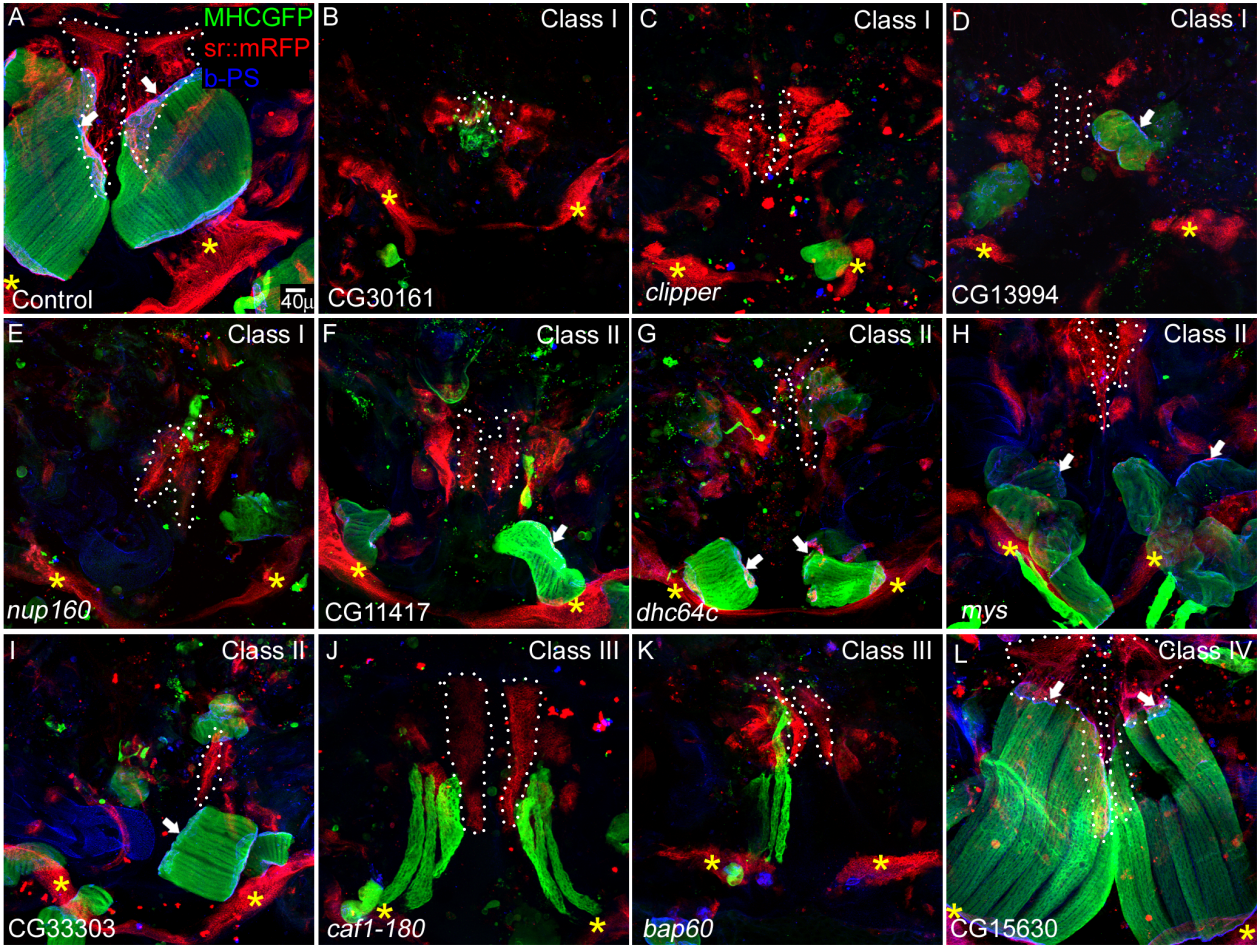
Myotendinous system phenotypes in tendon mediated RNAi knockdown at 36 h APF. Dorsal longitudinal muscles (DLMs) (MHC-tau-GFP, green) thoracic tendon cell clusters (*sr*-Gal4,UAS-myr mRFP, red) and myotendinous junction (β-PS integrin, blue) are shown at 36 h APF. (A) In control thoracic preparations, DLMs are attached to two tendon cluster anterior (dotted line) and posterior (yellow asterisk) and integrin accumulation is seen at the junction (anterior junction is shown with white arrow). Class I candidates (B-E) show severely affected tendon cell clusters (dotted line and yellow asterisk in B-D, white and yellow asterisk in E); DLMs are very small or absent. Class II candidates (F-I) show severe defect in tendon cells (dotted line and yellow asterisk in F, G, I and white and yellow asterisk in H,) similar to class I but, DLMs are small and attached to the posterior tendon cells (yellow asterisk) and show integrin accumulation at the anterior and posterior ends (anterior integrin accumulation is shown in white arrow). Class III candidates show developmental defect (J, K) wherein the thoracic preparations appear similar to an early staged preparation, tendon cells are marked with dotted line and yellow asterisk, larval templates are seen. Class IV candidates do not show any defect in myotendinous system (L) (n = 5).

During early pupal development larval templates grow in size by fusing with swarming myoblasts and send filopodial extensions to attachment sites [14]. Attachment sites for DLMs develop on the wing disc notum, which are specified by the expression of *stripe* (*sr*) (S1J Fig) [15,16]. In total there are five clusters of tendon cell precursors are present on wing disc namely Cluster a, b, c, d and posterior cluster (S1J Fig). The wing disc evaginates and these attachment sites are positioned near developing DLMs in second thoracic segment (S1J–S1K” Fig). DLMs attach to cluster ‘a’ at dorsal-anterior end and to posterior cluster at posterior end (Fig 1A, marked by dotted area and yellow asterisk). Proper specification and differentiation of myoblasts and tendon cells as well as co-ordinated MTJ formation are important to establish and maintain appropriate muscle structure and function. While several molecules are identified in myogenesis [17,18,19,20,21,22], mechanisms by which tendon cells and MTJs are specified and maintained are not completely understood. Hence we undertook a tendon cell specific genetic screen to identify novel genes that affect MTJ formation.

Using transgenic RNAi lines and the Gal4-UAS system [23], we selectively knocked down 1384 genes (2507 RNAi line) (S1 and S2 Tables) individually in tendon cells using tendon cell specific *stripe*-Gal4 (*sr*-Gal4) [24] and scored for lethality or flight defect as well as defective adult myogenesis. The RNAi lines targeting specific genes were selected in an unbiased manner in order to uncover novel candidate genes. Here, we report the identification of novel genes that affect MTJ formation. Further, we show that an endoplasmic reticulum (ER) to Golgi transport protein Tango1 (Transport and Golgi organization 1) is essential for proper development of tendon precursors and MTJ formation. We further show a role for Tango1 in secretion of the components of the extracellular matrix (ECM) from hemocytes and fat cells. Thus, our study provides a foundation for understanding the role of transport proteins in ECM secretion and MTJ maintenance along with novel molecules of tendon cell development and MTJs formation.

## Materials and Methods

### Fly stocks

*sr*-Gal4 [24], *mhc*-tau-GFP [13], *tubulin*Gal80^ts^(7108) and UAS-myr-mRFP(7119) (Bloomington Drosophila stock center), UAS-mannosidaseII GFP [25], *cg-*Gal4 [26], UAS-RNAi lines ([27] and NIG, Japan), UAS-dicer2[27], GS15095, GS17108 and GS21664 (DGRC), *viking*-GFP [28].

### Genetic screen

2507 UAS-RNAi lines were crossed to *sr-*Gal4,UAS-myr-mRFP,*mhc*-tau-GFP/TM6,Tb and screened for lethality or flight defect. Flies were reared at 25°C for screen. MTJs at 36 h APF/24 h after puparium formation (APF) stage were analyzed for the phenotypes. RNAi lines yielding embryonic lethality in combination with *sr-*Gal4,UASmyr-mRFP,*mhc*-tau-GFP/TM6,Tb were crossed to tubulinGal80^ts^/ tubulinGal80^ts^; *sr-*Gal4,UASmyr-mRFP,*mhc*-tau-GFP/TM6,Tb and reared at 18°C tills 2^nd^ instar larval/ 0 h APF stage before raising at 29°C to bypass early stage lethality.

### Pupal thoracic preparation and immunohistochemistry

White prepupae of 0 h age were picked and grown at 25°C (unless otherwise mentioned) to the desired stage. Pupal case was removed; pupae were dissected on Sylgard plate in 1X PBS (phosphate buffer saline) and fixed in 4% PFA (paraformaldehye) for 30 minutes. Samples were washed with 0.3% Triton-X100 in 1X PBS (PBT) and blocked with 0.3% BSA+0.3% Triton-X100 in 1X PBS (PBTB) for 45 minutes followed by overnight incubation with primary antibody (*see* Antibodies and Reagents) diluted in PBTB at 4°C. Primary antibody was removed and samples were washed with PBT, then incubated in secondary antibody diluted in PBT for 1.5 hours at room temperature, then washed with PBT and mounted in vectashield mounting medium (Vector Chemicals) [8].

### Larval body wall preparation

Wandering 3^rd^ instar larvae were picked and washed in double distilled water (ddH_2_O) and dissected on Sylgard plate in 1X PBS [29]. The body wall was preserved while the remaining tissues were discarded. The cleaned up body wall was subsequently processed for immunohistochemistry procedures as mentioned previously [8].

For 1^st^ instar larval body wall, newly hatched larvae were collected and dissected as mentioned above [29].

For fat cell preparations, dissection procedures were similar to the body wall preparation except, while mounting only fat cells were mounted [29].

### Hemocyte culture and immunohistochemistry

Larvae were washed serially in 1:1 ddH_2_O and bleach, 70% ethanol and ddH_2_O. Larvae were bled on Sylgard plate in 200μl of Schneider’s complete medium (SCM). SCM containing the bled larval contents was plated on a coverslip dish, incubated at 20°C or at room temperature for one hour, washed with 1X M1+BSA+Glucose and fixed in 2.5% PFA diluted in M1 for 20 minutes followed by 15 minutes permeabilization with 0.37% Igepal in M1. Subsequently, cells were washed in M1, blocked with 1X M1+BSA for one hour and incubated with primary antibody (diluted in 1X M1 + BSA) for one hour. After washing with M1, cells were incubated with secondary antibody (diluted in M1+BSA) for one hour, then washed with M1 and stained with Hoechst / DAPI (diluted in M1). The processed cells were used for imaging subsequently [30].

### Antibodies and reagents

Following antibodies and reagents were used in this study: Chicken anti-GFP (1:500), rabbit anti-RFP (1:500) and rabbit anti- GM130 (1:100) from Abcam, rabbit anti-GFP (1:10000), Rhodamine-labeled phalloidin (1:200) and DAPI (1:500) from Molecular Probes, Invitrogen, rabbit anti-dsred (1:500) from Clontech, mouse anti-βPS (1:20) from DSHB. Rabbit anti-Sparc (1:500) was a generous gift from Maurice Ringuette, University of Toronto, Canada, guinea pig anti-Tango1 (1:200) was a kind gift from Sally Horne-Badovinac, University of Chicago.

### Imaging and image processing

Samples were imaged using Olympus Fluoview1000, Zeiss510 or Zeiss700 microscopes. Images were processed using Olympus fluoview viewer, ImageJ and Adobe Photoshop softwares.

Calculation of the area of tendon cell cluster on wing disc was done using ImageJ and graph was plotted using Microsoft Excel.

### Time-lapse imaging

MTJ time-lapse imaging for DLMs has been previously described [14,31]. In our experiments, pupa of the correct age was directly transferred into a custom slide with a slit, without removal of the cuticle or any other perturbation. Olympus FV1000 was used for imaging, with Z-stacks being acquired every 4–10 minutes. The movies were made using Fiji (ImageJ). The orientation of pupae differed in different genotypes because of architectural differences in the developing animal inside cuticle.

## Results

### Temporal analysis of DLM MTJ formation

DLMs are indirect flight muscles in adult *Drosophila*, which develop on larval templates during pupal development. At 12 h APF, three larval templates are seen in each hemithorax (S1A Fig), which by 15 h APF initiate contact with developing tendon cells and start splitting longitudinally and yield to six DLM fibres by 18 h APF (S1B and S1C Fig and S1 Movie). The DLM’s interaction with target tendon cells further matures and MTJs are clearly visualised (S1D and S1E Fig). During early phase of MTJ formation, tendon cells extend filopodial processes, which make contact with migrating muscle fibres [14]. Establishment of the correct cognate interaction is followed by accumulation of adhesion molecules and matrix proteins, for example, β-PS integrin (S1H and S1I Fig) and Thrombospondin [32,33]. Muscles were visualized by MHC-tau-GFP and tendon cells with membrane targeted RFP driven by *sr-*GAL4. MTJs were visualized by an antibody against β-PS integrin (Fig 1A).

### An *in vivo* RNAi screen to identify genes involved in the formation of MTJs

We performed a genetic screen using a UAS-RNAi library from NIG, Japan and VDRC, Vienna stock centers. In the primary screen, 2507 UAS-RNAi lines spanning 1384 annotated genes were screened by crossing with tendon specific *sr*-Gal4 and scored for developmental lethality or flight defect as the defective MTJs could cause lethality or flight defect. Of the 21 candidates identified, 17 were pupal lethal, 3 were embryonic lethal and 1 showed inability to flight (Table 1). These candidates were subjected to a secondary screen, in which the animals carrying a single copy of *sr*-Gal4 and UAS-*RNAi* were examined at 24h or 36h APF for defects in the formation of the myotendinous system of DLMs.

**Table 1:** Classification of RNAi candidates based on known/predicted function.

Transcription factor	Enzyme activity	Chromatin remodelling	Intracellular transport/Secretion	Cell adhesion	Protein folding	Unknown
*bap60*	CG33303	*caf1-180*	*dhc64c*	*mys* *	*l(3)01239* ^#^	CG3124
CG7339	*clp*		*nup160*	*talin* *		CG11030
*ecr*			*tango1*			CG11417
*taf2*			*tango4* *			CG13994
*tfIIb*						CG15630
						CG30161

* Embryonic lethal.

^#^ flight defect, rest were lethal at pupal stages.

The knockdown screen yielded 21 candidates involved in various molecular functions (Table 1) such as intracellular transport, protein folding, cell adhesion, transcription factor activity, enzyme activity and chromatin remodeling. Six out of 21 candidates were of unknown function. Of the previously characterized genes, we identified *myospheroid* (Integrin-β*PS*) and *talin*, which are known to play a role in MTJ development [34,35,36,37,38], thus validating our screen. Other candidates identified in the screen have not been reported or investigated in the myotendinous system, particularly in tendon cell mediated regulation, thus they are novel regulators of myotendinous development.

### Phenotypic characterization and classification of candidate genes

Of the 21 candidates identified, 20 were further classified into four classes based on the severity of phenotypes of muscle and tendon cell development and MTJ formation (Table 2). DLMs attach to the two clusters of tendon cells, one at the dorsal-anterior end and other at the posterior end (Fig 1A) [15,39]. Candidates showing defective tendon cell clusters at both the attachment sites of DLMs were designated as class I. In class I candidates, both dorsal-anterior and posterior tendon cell cluster size is reduced (compare the cluster marked with white dotted line and yellow asterisk in Fig 1A and 1B–1E). These candidates also show severe defects in muscle development wherein the DLMs are either absent or reduced in size (Fig 1B–1E). Class II candidates also show severe phenotypes in the context of MTJ formation. In these candidates, tendon cluster size is reduced and muscle fibres are smaller, though the posterior tendon cell clusters show attachment with DLMs. MTJs at the dorsal-anterior attachment site are not seen in the thoracic preparations of these candidates at 36 h APF (Fig 1F–1I).

**Table 2:** Classification of candidates based on the knockdown phenotype with *sr*-Gal4.

Phenotype Class	Gene	Known/predicted function	Human ortholog#	Known Human Disease association
**Class I**	CG13994	Protein phosphatase inhibitor	PPP1R11	Yes
	CG30161	unknown	No ortholog	No
	*clp*	endoribonuclease	CPSF4/CPSF4L	Yes
	*nup160*	Nucleocytoplasmic transport	NUP160	Yes
**Class II**	CG11417	unknown	ESF1	No
	CG33303	Glycosyl transferase	RPN1	Yes
	*dhc64c*	Microtubule motor protein	DYNC1H1	Yes
	*mys*	Reception/adhesion	ITGB 1,2	Yes
	*tango1*	Cargo binding protein	CTAGE8/MIA3	Yes
	*tango4*	Pre-mRNA splicing	PLRG1	Yes
**Class III**	*bap60*	Transcription factor	SMARCD1,3	Yes
	*caf1-180*	Unknown	CHAF1A	Yes
	*ecr*	Hormone receptor	NR1H3,4	Yes
**Class IV**	CG11030	unknown	NGDN	No
	CG3124	unknown	No ortholog	No
	CG31970	Unknown	NCAM1	Yes
	CG7339	Transcription factor	POLR3H	No
	*l(3)01239*	Chaperon	PFDN2	Yes
	*taf2*	Transcription factor	TAF2	Yes
	*tfIIb*	Transcription factor	GTF2B	Yes

^**#**^ based on DRSC Integrative Ortholog Prediction Tool result.

A subset of candidates showed developmental delay where muscles at 36 h APF were comparable in size and morphology to those at 12–18 h APF in control thoracic preparations. These genes responsible for developmental timing defects were categorized in class III (Compare Fig 1J and 1K to S1A Fig). Candidates categorized in class IV were pupal lethal but did not show obvious phenotypes in the formation of myotendinous system of DLMs compared to controls (Fig 1L). Table 2 and S3 Table lists the human orthologs present for the candidates picked in the screen and their association with human diseases.

**Fig 2:**
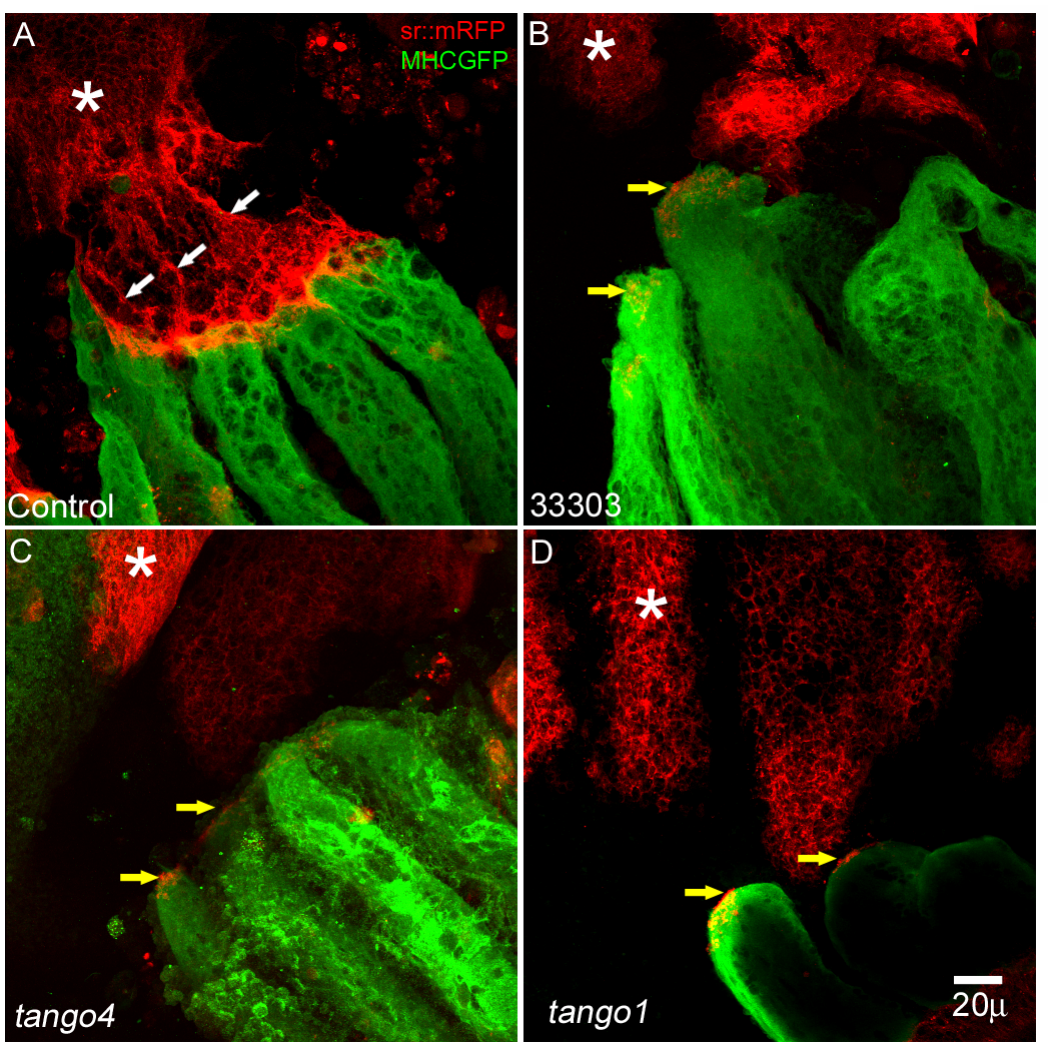
Myotendinous junction formation fails in knockdown of CG33303, Tango1 and Tango4. (A) In Control myotendinous system at 24 h APF, cognate interaction of DLMs and their attachment site (tendons) is clearly visible (DLMs in green, attachment sites in red), filopodial extension of tendon cells are seen (white arrows). (B-D) In CG33303, *tango4* and *tango1* RNAi animals, DLMs are detached from their attachment site and show red speckles at their anterior ends (yellow arrows). Tendon cells do not show filopodial extension in these knockdown animals. (n = 4), white asterisk mark the target attachment site for DLMs.

Class II candidates show interesting phenotype wherein only dorsal-anterior MTJ is affected. MTJ formation is already stabilized by 36h APF. To investigate whether the phenotypes observed are result of improper junction formation or failure of MTJ maintenance, we examined the MTJs of three of the class II representative candidates at an earlier time point of 24 h APF. While controls showed numerous cellular projections and cognate interactions with DLM fibres in the anterior tendon cell cluster (Fig 2A), CG33303, *tango1* and *tango4* knockdown preparations showed reduced cellular projections and DLM fibres were detached from the tendon cluster. As these fibres show red fluorescent speckles at their anterior end (Fig 2B–2D, yellow arrows), it is probable that the DLMs make contact with tendon cells, marked by red fluorescent protein, but fail to maintain a cognate interaction in order to form a stable junction. Hence, we chose a candidate, Tango1, with this phenotype for further analysis.

### Tango1, an ER exit protein identified as a candidate involved in MTJ formation

**Fig 3:**
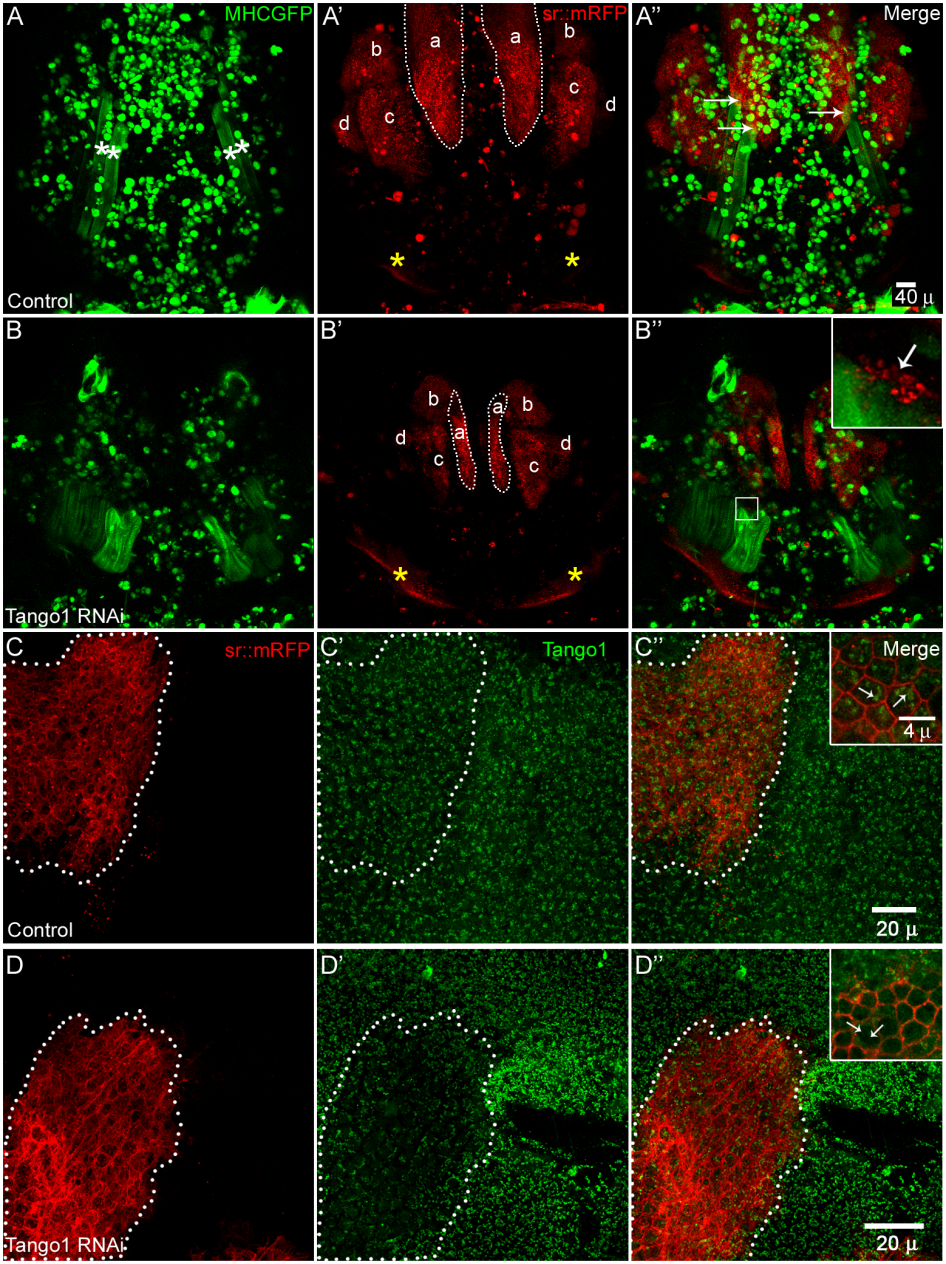
Tendon cell development and differentiation affected in Tango1 knockdown. (A-A”) live control animal at 24 h APF shows DLMs (white asterisk in A, only two are visible in either hemisegment) and tendon cell clusters (a, b, c, d and yellow asterisk, dotted line shows the anterior target attachment site and yellow asterisk shows posterior attachment site for DLMs). Cognate interaction of DLMs and tendon cells are shown in A” (white arrows). (B-B”) live *sr*>*tango1* RNAi animal at 24 h APF shows DLMs are not attached to its target tendon cell cluster at anterior end (dotted line, a) though it is attached to posterior cluster (yellow asterisk). Inset in B” shows the RFP speckles attached to the anterior end of DLMs. (C-C”‘) Tango1 staining in tendon cells is shown in control animal (in green, tendon cells are marked in red). (D-D”‘) Levels of Tango1 is low in *sr*>*tango1* RNAi tendon cells (compare dotted area in D’ with C’ and inset in D” to C”). Muscles are marked in green in A-B”, Tango1 staining is labelled in green in c-D” and tendon cells are shown in red in all panels. (n = 4 for A and B, n = 7 for C and D)

Tango1 (Transport and Golgi organization 1), a candidate gene classified into class II, was previously identified in an RNAi screen in S2 cell as an ER exit protein with an important role in ER to Golgi transport and Golgi organization [40]. Tango1 is known to be involved in secretion of Collagens in both vertebrates and invertebrates [28,41,42,43,44]. We confirmed the expression of Tango1 in tendon cells by immunohistochemistry using anti-Tango1 antibody. We further assessed the myotendinous phenotypes in tendon-specific Tango1 depletion. Tango1 knockdown in tendon cells was validated by Tango1 antibody staining of thoracic pupal preparation of *sr*-Gal4,UASmyr-mRFP,*mhc*-tau-GFP/UAS-*tango1*RNAi. (Fig 3C–3D”). Tango1 knockdown in tendon cells showed reduction in the size of tendon cell clusters (white dotted area in Fig 3A’ and 3B’) at 24 h APF. As tendon cell precursors are specified on the wing imaginal disc during 3^rd^ larval instar we looked at the tendon precursors in the control and *sr* mediated *tango1* knockdown. We found that the size of tendon cell cluster “a” on wing disc was smaller in the *tango1* knockdown animals in comparison to controls (S2 Fig).

By 24 h APF, wild type (*sr*-Gal4,UASmyr-mRFP,*mhc*-tau-GFP/+) MTJs of DLMs are already seen, whereas in tendon-specific *tango1* knockdown, the dorsal-anterior MTJ is not formed (Fig 3A” and 3B”). Intriguingly, DLMs show part of tendon cells attached to their ends (inset in 3B”) suggesting that there was initiation of contact between muscle and tendon cells but MTJ did not mature. To confirm the initiation of muscle tendon contact we performed time-lapse imaging of tendon specific *tango1* knockdown pupae without removing cuticle at various time windows viz. 15 h APF, 21.5 h APF, 23 h APF. We observed MTJ formation and attachment in all observed pupal stages (S2, S3 and S4 Movies). In the early pupa (S2 Movie), the myotubes extend projections towards the tendon cells, followed by attachment initiation and splitting (S3 and S4 Movies). We did not observed detachment of MTJ in the time lapse movies. While during very carefully done thoracic preparations muscles in all the tango1 knockdown animals are affected i.e. they are not attached to the tendon cell cluster. In case of control thoracic preparation muscle detachment because of the experimental error is very less (2 out of 11). This suggests that the MTJ formed in *tango1* knockdown animals are extremely weak and could break even with a very slight mechanical perturbation like removal of pupal case (Fig 3B”). However, in one case we have observed the detachment without any mechanical perturbations (S3 Fig). Also the morphology of the *tango1* knockdown pupae gets abrogated progressively which makes it difficult to study the details of myotendinous system.

Since tendon-specific Tango1 knockdown causes pupal lethality, we investigated whether Tango1 is essential before pupal development. The tendon cell-specific driver *sr*-Gal4 is known to be active in embryonic and larval stages. To increase the extent of depletion, we combined it with *dicer2* and found that UAS-dicer2/+;+/+;*sr*-Gal4,UASmyr-mRFP,*mhc*-tau-GFP/UAS-*tango1*-RNAi animals were lethal at late third instar larvae and showed cuticle detachment (S4 Fig). This suggests Tango1 is necessary in tendon cells at larval stage of development as well.

### Tango1 function is required for secretion of Collagen and BM-40 SPARC

Muscle and tendon cells secrete signaling molecules and ECM components during MTJ formation. Vein, an EGFR ligand is secreted by muscle cells and binds to its receptor on the tendon cells to activate EGFR signaling [45]. Secretion of ECM components, from tendon cells is a prerequisite for MTJ formation during *Drosophila* embryonic development [32]. Tango1 has a very important role in secretion and Golgi organization as described earlier. Our knockdown analysis suggests that Tango1 is required for ECM secretion at the MTJs. To understand the role of Tango1 in secretion of ECM components, we used hemocytes and fat body cells as a model. In particular, we analyzed the role of Tango1 in hemocytes as they are amenable to primary cell culture and hence better suited to cellular level analysis.

**Fig 4:**
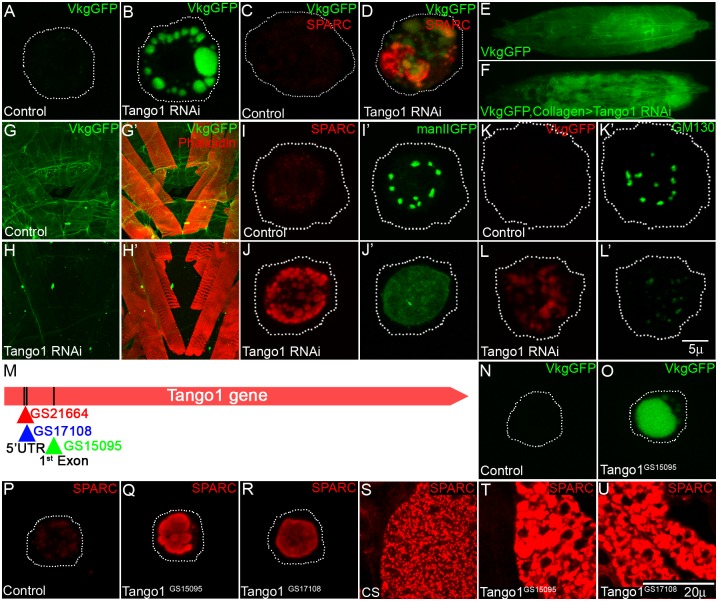
Tango1 is required for Golgi organization and secretion of Collagen and BM-40 (SPARC). (A-B) Tango1 knockdown in hemocytes show accumulation of VkgGFP (B, compare with A). (C-D) Sparc is accumulated in hemocytes of Collagen-specific Tango1 knockdown L3 larvae (D) in comparison to hemocytes of control L3 larvae (C). (E-F) VkgGFP L3 larvae shows GFP expression throughout larval body (E), GFP expression is not seen throughout in L3 larvae of Collagen specific Tango1 knockdown, but high intensity GFP speckles are seen in fat body (F). (G-H’) In control larval flat preparations (VkgGFP), Collagen (marked by VkgGFP in F) is deposited in basement membrane; muscles are marked with Rhodamine labelled phalloidin (G’) as counterstain. In Collagen-specific Tango1 knockdown, levels of Collagen (marked by VkgGFP in H) are low, muscle are shown in H’. (I-L’) Golgi organization in Tango1 knockdown, hemocytes is affected shown by mannosidase-II GFP (J’, compare with I’) and GM130 (L’, compare with K’). I and J show Sparc staining, K and L show Vkg GFP staining in hemocytes. (M) Tango1 gene locus showing GS insertions, GS15095 and GS17108 and GS21664 at its 5’ end. (N-O) Hemocytes from L1 staged VkgGFP show no accumulation of VkgGFP inside cell (N), hemocytes from GS15095 homozygous L1 VkgGFP is accumulated inside cell (O). (P-R) BM-40 Sparc, an ECM protein is accumulated in the hemocytes of lethal GS insertion, GS15095 (Q) and GS17108 (R); compare with P. (S-U) Sparc is accumulated in fat cells of lethal GS insertions, GS15095 (P) and GS17108 (Q); compare with O. Note: n>20 for hemocytes, n = 5 for fat cells and body wall preparations. H and I are taken at Olympus stereozoom microscope; all others were imaged in Laser scanning confocal microscope.

In *Drosophila*, hemocytes and fat cells are the major cell types involved in synthesis and secretion of many ECM components such as Collagen, SPARC (Secreted Protein Acidic and Rich in Cysteine), and Laminin [46,47]. We analyzed secretion in control and Tango1-depleted hemocytes and fat body cells by monitoring localization of Viking-GFP, a Collagen reporter and SPARC. Knockdown of Tango1 with *cg*-Gal4 (expressed in collagen synthesizing cells) showed retention of Collagen (as seen by Viking-GFP) and SPARC in hemocytes and fat cells as compared to controls (Fig 4A–4F; n = 119 cells, 57 percent cells show retention of Collagen inside cell). We confirmed the knockdown by immunohistochemistry with anti-Tango1 antibody (S5 Fig). The outcome was low levels of collagen in basement membrane surrounding larval tissues as seen by VikingGFP staining (Fig 4H–4H’ compared to 4G–4G’; n = 10, all preparations show low level of Collagen). Collagen and SPARC are known to interact with each other. DCg1^412^,a recessive lethal allele of Collagen type IV and a deficiency covering both the Collagen type IV genes show reduced levels of SPARC expression in embryonic hemocytes whereas in SPARC mutant embryos the basal lamina lacks Collagen [48,49]. Tango1 mediated secretion occurs via COPII coated vesicles wherein vesicles bud from ER and fuse with Golgi, also Tango1 knockdown is reported to affect Golgi organization [40,42,43]. Hence, we looked at Golgi organization in Tango1 knockdown to see if it was affected. The Golgi lumen marker mannosidase II-eGFP and Golgi membrane marker Golgi matrix 130 (GM130) were both aberrantly localized in Tango1 depleted cells (Fig 4I–4L’). Mannosidase-IIeGFP appeared diffused in contrast to its punctate appearance in control hemocytes suggesting a possibility of fusion of Golgi with the ER (Fig 4I–4J’). GM130 localized to punctae which were higher in number and lower in intensity compared to control, suggesting a redistribution of the Golgi membrane in mutant cells (Fig 4K–4L’).

To further validate the RNAi data, we analyzed three GS lines—GS15095, GS17108 and GS21664 carrying gene search element insertion [50] in the Tango1 locus and thus disrupting the *tango1* gene (Fig 4M). GS15095 insertion is present in the first exon, while the GS17108 and GS21664 insertions are present in the 5’ UTR region. All these insertions are homozygous lethal at first larval instar; two insertions, GS15095 and GS17108 showed lethality in trans-allelic combinations as well. We recombined GS15095 line with Viking-GFP and visualized the hemocytes for retention of Viking-GFP in wild type (Viking-GFP) and the recombined insertion (GS15095,Viking-GFP/GS15095). Hemocytes and fat cells isolated from GS15095 and GS17108 homozygous larvae retain collagen (Viking-GFP) (Fig 4N and 4O) and SPARC (Fig 4P–4R and 4S–4U). This confirms the role of Tango1 in secretion of ECM-components.

## Discussion

Several molecular players are conserved in vertebrates and insect MTJ formation. Perturbation of gene expression for molecules that affect development of adult muscle, tendons or MTJs is expected to result in lethality or aberrant structure and function of myotendinous system. MEF2, a regulator of myogenesis, is important for the differentiation of muscles in vertebrates as well as in *Drosophila* [51,52]. *stripe* (*sr*), an early growth response-like zinc finger transcription factor is necessary for the differentiation of tendon cells and its vertebrate homologs EGR1 and EGR2 play an important role in tendon cell differentiation [15,16,53,54,55]. *Drosophila* Thrombospondin and its vertebrate homolog are both shown to be involved in development of MTJ [32,33,56].

In this study we identified several molecules that affect *Drosophila* MTJ formation. In spite of the small number of genes analyzed, our tendon-specific screen yielded known and new players in MTJ formation or maintenance. Expectedly, these cover a wide range of molecular functions including intracellular transport/secretion, ECM deposition, transcription, chromatin-remodelling and enzyme activity. The four phenotypic classes that we identified ranged from severe to mild phenotypes in context of DLMs and their attachment sites. Knockdown of Class I genes *clipper*, *nup160*, CG13994 and CG30161 resulted in drastically reduced anterior and posterior tendon clusters, reduced or absent muscle and failure of MTJ attachment indicating a role in early development of the adult thoracic tendon clusters. There is a possibility that these molecules could affect the tendon cell precursor development on wing disc. Clipper is an endoribonuclease and Nup160 is involved in nucleocytoplasmic transport. Functions of CG13994 and CG30161 are not known. Clipper, a homolog of human CPSF4, is a component of cleavage and polyadenylation complex necessary for 3’ mRNA processing. It is known that the unprocessed mRNAs are not exported from the nucleus [57]. Nup160 is a subunit involved in formation of nucleoporin complex; it has been shown to play role in SMAD nuclear transport and mRNA export [58,59]. Furthermore, it is shown to be involved in microtubule polymerization [60]. In *Drosophila* tendon cell development, nuclear export of the *stripe* mRNA is of particular importance [61]. Similarly these molecules could be regulating the nuclear export of the mRNA necessary for the development and differentiation of tendon cells. The severe muscle phenotype in this class of molecules could be due of lack of interaction between the developing tendon cells and the developing myotubes.

Class II candidate knockdown also show a severe phenotype. In these cases, the dorsal-anterior MTJ is severely defective where we did not see any stabilized junction, whereas the posterior attachment site remains less affected. This class includes *dhc64C*, *mys*, *tango1*, *tango4*, CG11417 and CG33303. *dhc64C*, *tango1* and *tango4* are involved in intracellular transport/secretion [40,42,62], *mys* is involved in cell adhesion and signaling [63,64,65,66,67], CG33303 has been predicted to have enzymatic activity while the function of CG11417 is not known. Dhc64C, a microtubule motor protein homologous to human cytoplasmic dynein heavy chain1, has been shown to interact genetically with EGF receptor and EGF ligand trafficking [68]. *mys* gene codes for integrin beta-PS protein which has been extensively studied and its role in development of myotendinous system is well established in *Drosophila* as well as vertebrates [32,56,69,70,71]. It interacts with other integrin subunits to form the heterodimers which interact with the components of extracellular matrix. Tango1 and Tango4 are involved in cell secretion and Golgi organization [40]. Tango1 has been shown to interact with COPII complex and regulate ER to Golgi transport [42]. Tango4 is orthologous to human pleotropic regulator 1 gene (PLRG1) which is predicted to have mRNA splicing function [72]. The function of CG11417 is not known but it shows similarity with human nucleolar pre-RNA processing protein ESF1. CG33303 is predicted to have the dolichyl-diphosphooligosachharide-protein glycotransferase activity based on its similarity with human Ribophorin I. As candidates from this class show differential effect on different tendon cell clusters i.e. dorsal-anterior and posterior tendon cell cluster it would be interesting to investigate their role in specifying these tendon cell clusters.

A phenotype of developmental delay during early pupal stage is observed in class III mutants as these animals die during late pupal development. Candidates in this class are bap60, caf1-180 and ecr. Bap60, an essential component of Brahma complex, is known to bind DNA and is predicted to function as a transcription factor; it also has similarity to the human SMARCD1 and SMARCD2 [73]. Caf1-180 codes for the chromatic assembly factor subunit; it is predicted to be involved in nucleosome assembly based on mutant phenotype and its similarity with human CHAF1A [74]. *ecr* codes for ecdysteroid hormone receptor (EcR), which acts as ligand activated transcription factor. EcR functions in an array of biological processes throughout development of the *Drosophila* [75,76,77]. Interestingly the other two genes in this class are related to chromatin remodelling, and chromatin remodelling factors are implicated in the ecdysone mediated expression of genes [78,79]. The phenotype showing the delay in development of animals could be because of the expression of *sr*-Gal4 in other tissues like tracheal cells and central nervous system [80].

Class IV candidates had no apparent phenotype at DLM’s MTJ but were pupal lethal except one which showed flight defect suggesting that these may not have direct relevance to DLM MTJ formation. The candidates in this class are *taf2*, *tfIIb*, CG7339, *l(3)01239*, CG3124, CG11030 and CG31970. Taf2 and TfIIb are transcription factors associated with RNA polymerase II complex [81,82] whereas CG7339 is predicted to function as component of RNA polymerase III complex based on its similarity with *Saccharomyces* RPC25. The *l(3)01239* gene product is predicted to have a chaperon binding function based on its similarity with human PFDN2. CG3124, CG11030 and CG31970 do not have any known/predicted function. The absence of any apparent phenotype at the DLM MTJ could be because of various possible reasons. There could have been low levels of expression of these genes which could have been sufficient to not show any defect at the DLM MTJ. There could still be defects at the other MTJs which have not been looked at in this study. Yet another possibility for the lethality phenotype could be attributed to the expression of Gal4 in other tissues as mentioned previously.

We identified a specific role for Tango1 in this process. Tango1 interacts with Collagen via the SH3 domain and facilitates loading of Collagen at the ER into vesicles [42]. Tango1 knockout mice are defective in the secretion of various collagens, including Collagen I, II, III, IV, VII, and IX, from chondrocytes, endothelial cells, fibroblasts and mural cells and there is defect in composition of extracellular matrix components [44]. In *Drosophila* fat cells and follicle cells, Tango1 is required for the secretion of Collagen [28,41]. We find that Tango1 is necessary for the proper formation and stabilization of DLM’s MTJ. The dorsal-anterior attachments are very weak as these attachments are formed at an early stage but a small mechanical perturbation could affect the stability of junction. Also as the dorsal-anterior tendon cluster size is smaller in *tango1* knockdown; it is possible that the cluster is not able to provide the required strength as compared to control tendon cell cluster. A defect in the size of dorsal-anterior cluster on wing imaginal disc suggests a role of tango1 in early development of tendon cells on the imaginal disc. We confirm the role of Tango1 in collagen secretion and find its role in secretion of another extracellular matrix component SPARC (BM-40). Collagen and SPARC are known to interact with each other, thus we do not rule out the possibility of effect of their interaction in the tango1 knockdown as well however, role of Tango1 in loading Collagen in the vesicles has been shown earlier [42,48,49]. Though the cargo of Tango1 in tendon cells is yet to be identified, we show that Tango1 is necessary for the development of tendon cells from an early stage and also needed for stable MTJ formation

The candidates picked in this screen have human orthologs which are associated with various different human diseases ranging from carcinomas to myopathies and Alzheimer’s. In the light of the orthologous similarity with human genes these candidates can be explored not only in myotendinous system but also in other cellular contexts.

## Supporting Information

S1 Fig Developmental profile of DLMs and their attachment site at early pupal stage. (A-E) Dorsal longitudinal muscle (green) and tendon cells (red) at 12, 15, 18, 21 and 36 h APF. DLMs develop on three larval templates (marked in green, A) in each hemisegment, split into six (white arrow in B) and migrate to attach to its target tendon cell cluster (marked by white asterisk in A-E). (F-I) Muscle tendon junction (MTJ) at 36 h APF, DLMs are marked with MHC-tau-GFP (F), attachment sites are marked with *sr*-Gal4,UASmyr mRFP (G) β-PS accumulation at MTJ is shown in blue channel (white arrow in H and I). I is merge. Boxed area in F and G are shown at high magnification in F’ and G’ respectively. Differentiated DLMs are seen in F’ marked with MHC-tau-GFP, columnar nuclear arrangement is seen marked by white arrow. (G’) tendon cells processes are seen marked by *sr*-Gal4,UASmyr-mRFP (white arrows in G’). (n = 5)
(TIF)

S2 Fig Tendon precursors on the wing disc are affected in *sr*-Gal4 mediated *tango1* knockdown animals. (A-A”) Control tendon cell precursors on wing disc marked with the *sr*-Gal4,UASmyr mRFP (different clusters are marked by a, b, c and d). (B-B”) The tendon cell precursor cluster a is reduced in size in *sr*-Gal4,UASmyr mRFP/UAS-*tango1* RNAi animals (compare dotted area in B with A). (C) A box plot showing difference between the cluster ‘a’ size in control and *tango1* knockdown animals (pValue = 0.0055). (n = 9 for A and 7 for B)
(TIF)

S3 Fig Muscle detachment in the live prep of tendon specific *tango1* knockdown. (A) A 27 h APF live pupae of *sr*-Gal4,UASmyr mRFP,*mhc*-tau-GFP/UAS-*tango1* RNAi raised at 29°C (to increase RNAi penetrance) shows muscle detachment from dorsal-anterior tendon cells (white asterisks). One of the muscle fibres still show attachment with tendon cells (yellow asterisk). Please note in this case cuticle was not removed from the pupae and thus no mechanical perturbation are there still we see detachment. This is the only pupae showing this phenotype without removal of pupal case.
(TIF)

S4 Fig Tendon-specific *tango1* knockdown shows cuticle detachment at larval stage. A) A wild type third instar larvae, body cuticle appears attached to the mesodermal layer. B) Cuticle (white asterisk) is detached from the mesodermal layer (marked with arrows) in sr> *tango1* RNAi + *dicer2*. (n = 5)
(TIF)

S5 Fig Tango1 RNAi convincingly knockdown Tango1 expression as shown by anti-Tango1 staining. (A-A”) Tango1 staining in the hemocyte cells of lymph gland tissue (CollagenGal4 expression is seen in GFP). (B-B”) Levels of Tango1 are low in the CollagenGal4 mediated *tango1* RNAi hemocytes (compare dotted area in B’ with A’) (n = 6). CollagenGal4 positive cells are marked in green, Tango1 is marked in red and nuclei are marked with Dapi in blue.
(TIF)

S1 Movie Stabilization of DLM-tendon junction after the initial contact between myotubes and their cognate tendon cell cluster. The developing dorsal-anterior tendon cell cluster (red) interacts with two of the developing DLM myotubes (green) in the *sr*-Gal4,UASmyr-mRFP,*mhc*-tau-GFP/+ pupa. The anterior end of the GFP labelled DLM myotube is already attached (indicated by yellow arrowheads) to the RFP labelled tendon cell cluster. The extensions from the myotube gradually shorten as the MTJ stabilizes. The movie was recorded from ~18hr 30min APF for 3hr 24min. Z-stacks were recorded every 5 min for the first ~1hr and then every 4 min for the rest of the imaging session. The movie is being played at 5 fps. Time is indicated in hr:min. Scale bar = 15μm.
(MOV)

S2 Movie Initiation of MTJ formation is observed in tendon-specific Tango1 downregulated animals. Animals with tendon-specific Tango1 knockdown (*sr*-Gal4,UASmyr-mRFP,*mhc*-tau-GFP/UAS-*tango1*-RNAi) initiates DLM-dorsal-anterior tendon attachment. The anterior extensions of the myotube (green) displays directed movement towards the dorsal-anterior tendon cell cluster (red). The white arrow indicates initial interaction between the DLM myotube and the tendon cells. The movie was recorded from ~15hr APF every 7 mins for ~6 hr. The movie is being played at 5 fps. Time is indicated in hr:min. Scale bar = 20μm.
(MOV)

S3 Movie MTJ formation is observed in tendon-specific Tango1 downregulated animals. In the animals with tendon-specific Tango1 knockdown (*sr-Gal4*,*UASmyr-mRFP*,*mhc-tau-GFP/UAS-tango1-RNAi*), the anterior end of the GFP labeled DLM forms attachment with the dorsal-anterior tendon cell cluster (red). The pupa was recorded from ~20hr30min APF every 10 mins for 4hr40min. Due to gradual drift, the focus was shifted at 02:20, which results in a slight shift of the frame. The movie is being played at 5 fps. Time is indicated in hr:min. Scale bar = 15μm.
(MOV)

S4 Movie Complete splitting of DLMs occurs in tendon-specific Tango1 downregulated animals. DLMs in the pupae with tendon-specific Tango1 knockdown (*sr*-Gal4,UASmyr-mRFP,*mhc*-tau-GFP/UAS-*tango1*-RNAi) undergoes complete longitudinal splitting. The entire span of the GFP labelled DLMs are seen attached to the RFP labelled tendon cell clusters. The arrows indicate the MTJs formed at the both ends of the myotube. The movie was recorded from ~21hr30mins APF every 7 min for ~100 min. The movie is being played at 5 fps. Time is indicated in hr:min. Scale bar = 25μm.
(MOV)

S1 Table List of UAS-RNAi lines from NIG, Japan screened using tendon specific sr-Gal4. (XLSX)

S2 Table List of UAS-RNAi lines from VDRC, Vienna screened using tendon specific sr-Gal4. (XLSX)

S3 Table Disease association with human orthologs of the candidates from RNAi screen. (DOCX)
