# Supplementary figures and images for: A Tendon Cell Specific RNAi Screen Reveals Novel Candidates Essential for Muscle Tendon Interaction
Source: PLoS One. 2015 Oct 21;10(10):e0140976. doi: 10.1371/journal.pone.0140976 (PMC4619581; doi:10.1371/journal.pone.0140976)

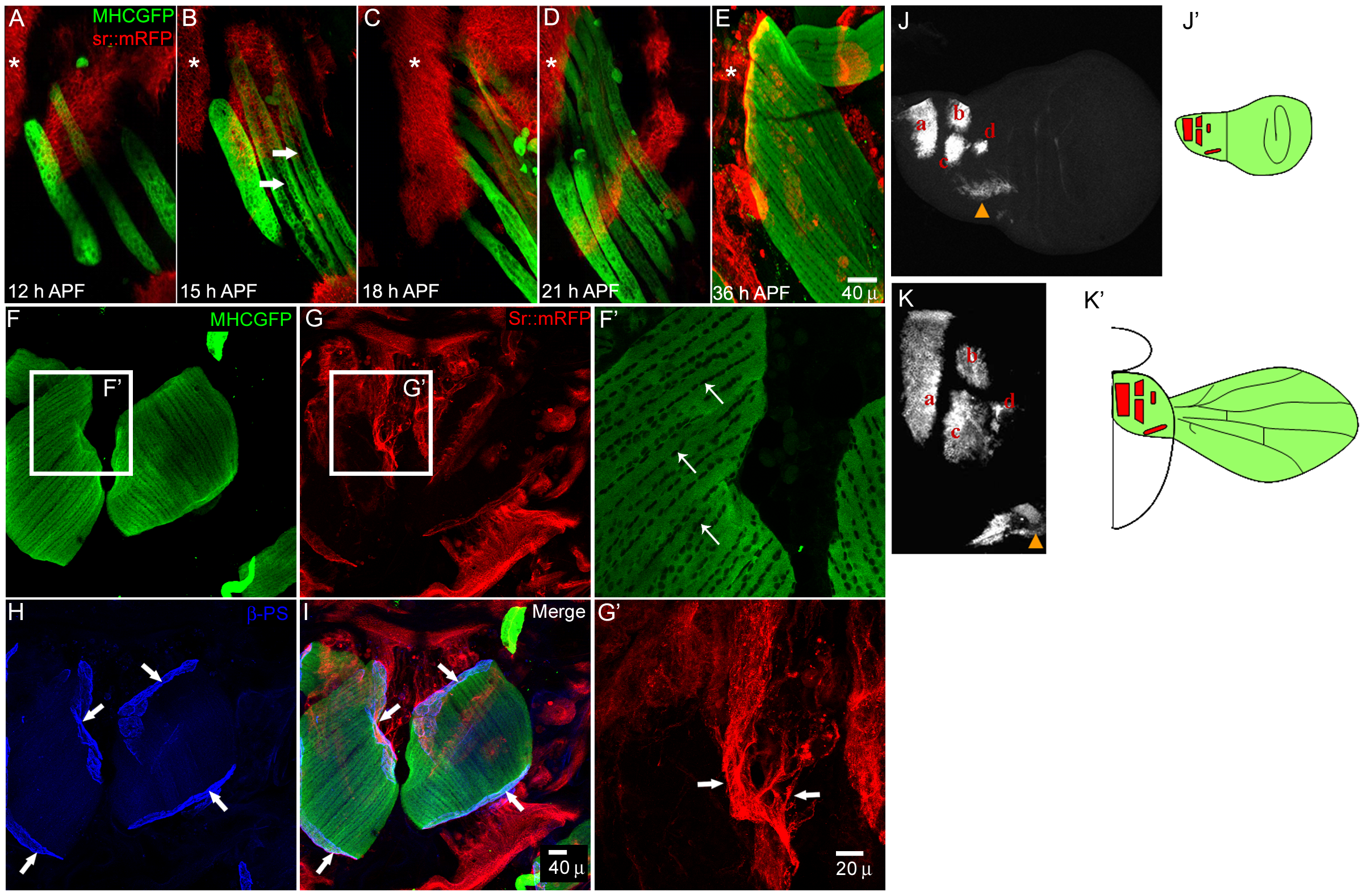

Supplement: S1 Fig — (A-E) Dorsal longitudinal muscle (green) and tendon cells (red) at 12, 15, 18, 21 and 36 h APF. DLMs develop on three larval templates (marked in green, A) in each hemisegment, split into six (white arrow in B) and migrate to attach to its target tendon cell cluster (marked by white asterisk in A-E). (F-I) Muscle tendon junction (MTJ) at 36 h APF, DLMs are marked with MHC-tau-GFP (F), attachment sites are marked with sr-Gal4,UASmyr mRFP (G) β-PS accumulation at MTJ is shown in blue channel (white arrow in H and I). I is merge. Boxed area in F and G are shown at high magnification in F’ and G’ respectively. Differentiated DLMs are seen in F’ marked with MHC-tau-GFP, columnar nuclear arrangement is seen marked by white arrow. (G’) tendon cells processes are seen marked by sr-Gal4,UASmyr-mRFP (white arrows in G’). (n = 5) (TIF) [file pone.0140976.s001.tif]

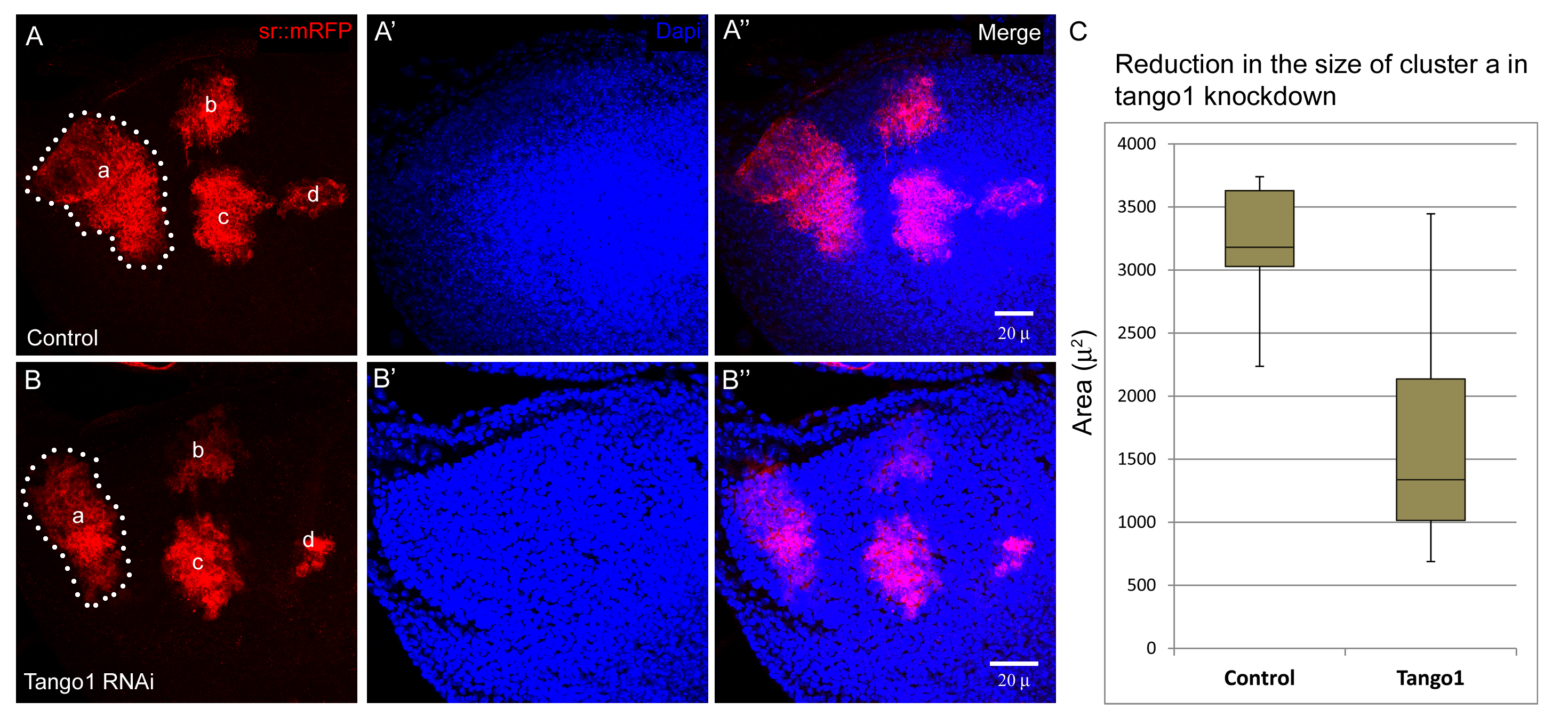

Supplement: S2 Fig — (A-A”) Control tendon cell precursors on wing disc marked with the sr-Gal4,UASmyr mRFP (different clusters are marked by a, b, c and d). (B-B”) The tendon cell precursor cluster a is reduced in size in sr-Gal4,UASmyr mRFP/UAS-tango1 RNAi animals (compare dotted area in B with A). (C) A box plot showing difference between the cluster ‘a’ size in control and tango1 knockdown animals (pValue = 0.0055). (n = 9 for A and 7 for B) (TIF) [file pone.0140976.s002.tif]

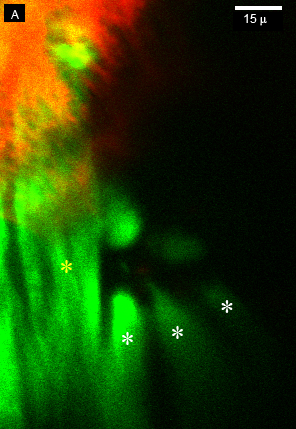

Supplement: S3 Fig — (A) A 27 h APF live pupae of sr-Gal4,UASmyr mRFP,mhc-tau-GFP/UAS-tango1 RNAi raised at 29°C (to increase RNAi penetrance) shows muscle detachment from dorsal-anterior tendon cells (white asterisks). One of the muscle fibres still show attachment with tendon cells (yellow asterisk). Please note in this case cuticle was not removed from the pupae and thus no mechanical perturbation are there still we see detachment. This is the only pupae showing this phenotype without removal of pupal case. (TIF) [file pone.0140976.s003.tif]

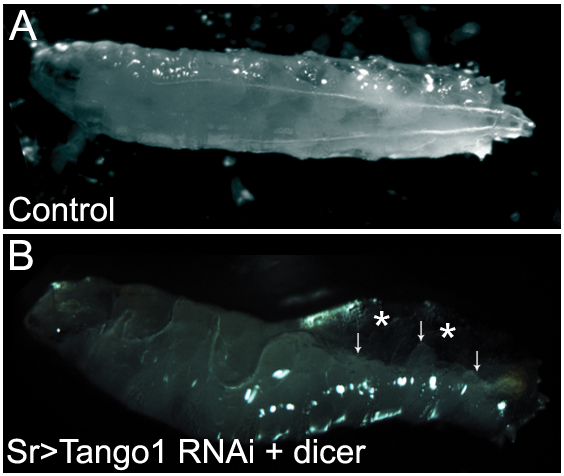

Supplement: S4 Fig — A) A wild type third instar larvae, body cuticle appears attached to the mesodermal layer. B) Cuticle (white asterisk) is detached from the mesodermal layer (marked with arrows) in sr> tango1 RNAi + dicer2. (n = 5) (TIF) [file pone.0140976.s004.tif]

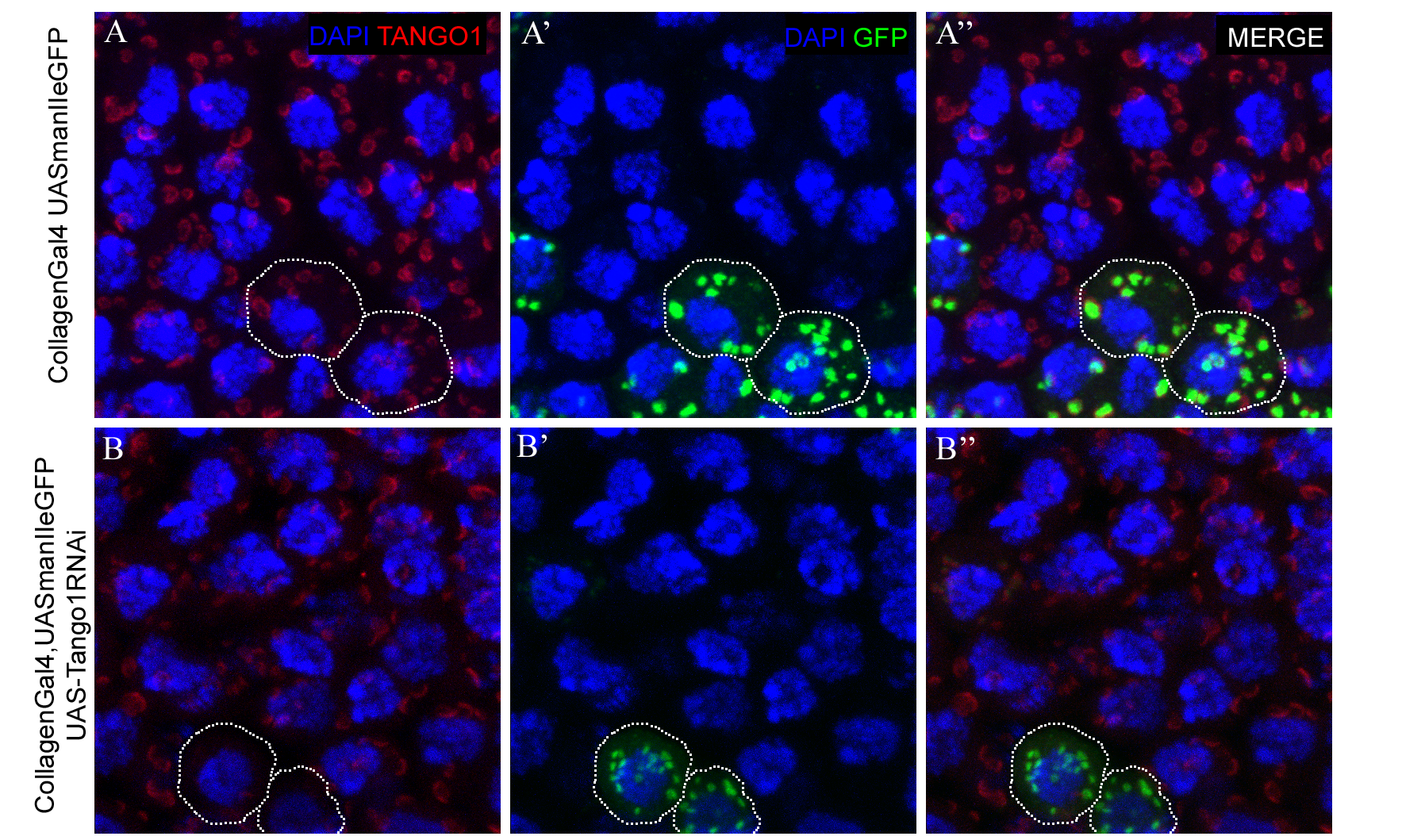

Supplement: S5 Fig — (A-A”) Tango1 staining in the hemocyte cells of lymph gland tissue (CollagenGal4 expression is seen in GFP). (B-B”) Levels of Tango1 are low in the CollagenGal4 mediated tango1 RNAi hemocytes (compare dotted area in B’ with A’) (n = 6). CollagenGal4 positive cells are marked in green, Tango1 is marked in red and nuclei are marked with Dapi in blue. (TIF) [file pone.0140976.s005.tif]
