# Supplementary material for: A Tendon Cell Specific RNAi Screen Reveals Novel Candidates Essential for Muscle Tendon Interaction
Source: PLoS One. 2015 Oct 21;10(10):e0140976. doi: 10.1371/journal.pone.0140976 (PMC4619581; doi:10.1371/journal.pone.0140976)
Supplement: S3 Table — (DOCX) [file pone.0140976.s012.docx]

# Disease association with human orthologs of the candidates from RNAi screen

| **Gene (Human Ortholog)** | **Human Disease associated (Reference)** |
| --- | --- |
| CG13994(PPP1R11) | Psoriatic arthritis[1] systemic Lupus Erythematosus[2] |
| CG30161(No ortholog) | -- |
| *clp*(CPSF4/CPSF4L) | lung adenocarcinoma[3] Influenza[4] |
| *nup160*(NUP160) | Ischemia and dilated cardiomyopathy[5] schizophrenia[6] |
| CG11417(ESF1) | -- |
| CG33303(RPN1) | Leukemia[7] |
| *dhc64c*(DYNC1H1) | Spinal muscular Atrophy[8] Charcot-Marie-Tooth disease, type 2O[9] |
| *mys*(ITGB 1,2) | Ischemic cardiomyopathy[10] human colorectal cancer[11] papillary thyroid cancer[12] |
| *tango1*(CTAGE8/MIA3) | Squamous cell carcinoma[13] Malignant melanoma[14] Colon and hepatocellular carcinoma[15] Myocardial infarction[16] Coronary artery disease[17] |
| *tango4*(PLRG1) | Poikiloderma with Neutropenia[18] |
| *bap60*(SMARCD1,3) | Breast Cancer[19] splenasthenic syndrome[20] |
| *caf1-180*(CHAF1A) | Neuroblastoma[21] |
| *ecr*(NR1H3,4) | Alzheimer's disease[22] osteosarcoma[23] hepatocellular carcinoma [24,25] |
| CG11030(NGDN) | -- |
| CG3124(No ortholog) | -- |
| CG31970(NCAM1) | Cardiomyopathy[26] multiple sclerosis[27] |
| CG7339(POLR3H) | -- |
| *l(3)01239*(PFDN2) | Bladder cancer[28] Huntington disease[29] |
| *taf2*(TAF2) | Microcephaly syndrome[30] |
| *tfIIb*(GTF2B) | Human hepatocellular carcinoma[31] |

1. Rahman P, Roslin NM, Pellett FJ, Lemire M, Greenwood CM, et al. (2011) High resolution mapping in the major histocompatibility complex region identifies multiple independent novel loci for psoriatic arthritis. Ann Rheum Dis 70: 690-694.

2. Barcellos LF, May SL, Ramsay PP, Quach HL, Lane JA, et al. (2009) High-density SNP screening of the major histocompatibility complex in systemic lupus erythematosus demonstrates strong evidence for independent susceptibility regions. PLoS Genet 5: e1000696.

3. Chen W, Guo W, Li M, Shi D, Tian Y, et al. (2013) Upregulation of cleavage and polyadenylation specific factor 4 in lung adenocarcinoma and its critical role for cancer cell survival and proliferation. PLoS One 8: e82728.

4. Kuo RL, Krug RM (2009) Influenza a virus polymerase is an integral component of the CPSF30-NS1A protein complex in infected cells. J Virol 83: 1611-1616.

5. Tarazon E, Rivera M, Rosello-Lleti E, Molina-Navarro MM, Sanchez-Lazaro IJ, et al. (2012) Heart failure induces significant changes in nuclear pore complex of human cardiomyocytes. PLoS One 7: e48957.

6. Camargo LM, Collura V, Rain JC, Mizuguchi K, Hermjakob H, et al. (2007) Disrupted in Schizophrenia 1 Interactome: evidence for the close connectivity of risk genes and a potential synaptic basis for schizophrenia. Mol Psychiatry 12: 74-86.

7. Pekarsky Y, Rynditch A, Wieser R, Fonatsch C, Gardiner K (1997) Activation of a novel gene in 3q21 and identification of intergenic fusion transcripts with ecotropic viral insertion site I in leukemia. Cancer Res 57: 3914-3919.

8. Tsurusaki Y, Saitoh S, Tomizawa K, Sudo A, Asahina N, et al. (2012) A DYNC1H1 mutation causes a dominant spinal muscular atrophy with lower extremity predominance. Neurogenetics 13: 327-332.

9. Weedon MN, Hastings R, Caswell R, Xie W, Paszkiewicz K, et al. (2011) Exome sequencing identifies a DYNC1H1 mutation in a large pedigree with dominant axonal Charcot-Marie-Tooth disease. Am J Hum Genet 89: 308-312.

10. Pfister R, Acksteiner C, Baumgarth J, Burst V, Geissler HJ, et al. (2007) Loss of beta1D-integrin function in human ischemic cardiomyopathy. Basic Res Cardiol 102: 257-264.

11. Song J, Zhang J, Wang J, Cao Z, Guo X, et al. (2014) beta1 integrin modulates tumor growth and apoptosis of human colorectal cancer. Oncol Rep 32: 302-308.

12. Eun YG, Kim SK, Chung JH, Kwon KH (2013) Association study of integrins beta 1 and beta 2 gene polymorphism and papillary thyroid cancer. Am J Surg 205: 631-635.

13. Sasahira T, Kirita T, Yamamoto K, Ueda N, Kurihara M, et al. (2014) Transport and Golgi organisation protein 1 is a novel tumour progressive factor in oral squamous cell carcinoma. Eur J Cancer 50: 2142-2151.

14. Arndt S, Bosserhoff AK (2006) TANGO is a tumor suppressor of malignant melanoma. Int J Cancer 119: 2812-2820.

15. Arndt S, Bosserhoff AK (2007) Reduced expression of TANGO in colon and hepatocellular carcinomas. Oncol Rep 18: 885-891.

16. Koch W, Schatke A, Wolferstetter H, Mueller JC, Schomig A, et al. (2011) Extended evidence for association between the melanoma inhibitory activity 3 gene and myocardial infarction. Thromb Haemost 105: 670-675.

17. Li X, Huang Y, Yin D, Wang D, Xu C, et al. (2013) Meta-analysis identifies robust association between SNP rs17465637 in MIA3 on chromosome 1q41 and coronary artery disease. Atherosclerosis 231: 136-140.

18. Shchepachev V, Wischnewski H, Missiaglia E, Soneson C, Azzalin CM (2012) Mpn1, mutated in poikiloderma with neutropenia protein 1, is a conserved 3'-to-5' RNA exonuclease processing U6 small nuclear RNA. Cell Rep 2: 855-865.

19. Stephens PJ, Tarpey PS, Davies H, Van Loo P, Greenman C, et al. (2012) The landscape of cancer genes and mutational processes in breast cancer. Nature 486: 400-404.

20. Ze-Min Y, Wei-Wen C, Ying-Fang W (2013) [Research on differentially expressed genes related to substance and energy metabolism between healthy volunteers and splenasthenic syndrome patients with chronic superficial gastritis]. Zhongguo Zhong Xi Yi Jie He Za Zhi 33: 159-163.

21. Barbieri E, De Preter K, Capasso M, Chen Z, Hsu DM, et al. (2014) Histone chaperone CHAF1A inhibits differentiation and promotes aggressive neuroblastoma. Cancer Res 74: 765-774.

22. Natunen T, Martiskainen H, Sarajarvi T, Helisalmi S, Pursiheimo JP, et al. (2013) Effects of NR1H3 genetic variation on the expression of liver X receptor alpha and the progression of Alzheimer's disease. PLoS One 8: e80700.

23. Chang YW, Zhao YF, Cao YL, Gu XF, Li ZQ, et al. (2013) Liver X receptor alpha inhibits osteosarcoma cell proliferation through up-regulation of FoxO1. Cell Physiol Biochem 32: 180-186.

24. Hu C, Liu D, Zhang Y, Lou G, Huang G, et al. (2014) LXRalpha-mediated downregulation of FOXM1 suppresses the proliferation of hepatocellular carcinoma cells. Oncogene 33: 2888-2897.

25. Su H, Ma C, Liu J, Li N, Gao M, et al. (2012) Downregulation of nuclear receptor FXR is associated with multiple malignant clinicopathological characteristics in human hepatocellular carcinoma. Am J Physiol Gastrointest Liver Physiol 303: G1245-1253.

26. Nagao K, Sowa N, Inoue K, Tokunaga M, Fukuchi K, et al. (2014) Myocardial expression level of neural cell adhesion molecule correlates with reduced left ventricular function in human cardiomyopathy. Circ Heart Fail 7: 351-358.

27. Gnanapavan S, Ho P, Heywood W, Jackson S, Grant D, et al. (2013) Progression in multiple sclerosis is associated with low endogenous NCAM. J Neurochem 125: 766-773.

28. Lopez V, Gonzalez-Peramato P, Suela J, Serrano A, Algaba F, et al. (2013) Identification of prefoldin amplification (1q23.3-q24.1) in bladder cancer using comparative genomic hybridization (CGH) arrays of urinary DNA. J Transl Med 11: 182.

29. Tashiro E, Zako T, Muto H, Itoo Y, Sorgjerd K, et al. (2013) Prefoldin protects neuronal cells from polyglutamine toxicity by preventing aggregation formation. J Biol Chem 288: 19958-19972.

30. Hellman-Aharony S, Smirin-Yosef P, Halevy A, Pasmanik-Chor M, Yeheskel A, et al. (2013) Microcephaly thin corpus callosum intellectual disability syndrome caused by mutated TAF2. Pediatr Neurol 49: 411-416 e411.

31. Li L, Zhang A, Cao X, Chen J, Xia Y, et al. (2013) General transcription factor IIb overexpression and a potential link to proliferation in human hepatocellular carcinoma. Pathol Oncol Res 19: 195-203.
